# A novel gene network analysis in liver tissues of diabetic rats in response to resistant starch treatment

**DOI:** 10.1186/s40064-015-0873-2

**Published:** 2015-03-05

**Authors:** Zhiwei Wang, Yinghui Zhang, Runge Shi, Zhongkai Zhou, Fang Wang, Padraig Strappe

**Affiliations:** Key Laboratory of Food Nutrition and Safety, Ministry of Education, Tianjin University of Science and Technology, Tianjin, 300457 China; School of Biomedical Sciences, Charles Sturt University, WaggaWagga, NSW 2678 Australia; School of Food Engineering and Biotechnology, Tianjin University of Science and Technology, Tianjin, 300457 China

**Keywords:** Cluster analysis, Gene network, Metabolism, Resistant starch, Diabetes mellitus

## Abstract

In this study, we investigated the genome-wide gene expression profiles in the liver tissue of diabetic rats before and after RS treatment. The microarray-based analysis revealed that a total of 173 genes were up-regulated and 197 genes were down-regulated in response to RS treatment. These genes were mainly related to glucose metabolism (e.g., hexokinase, pyruvate kinase and phosphotransferase etc.), and lipid metabolism (e.g., carnitine palmitoyl transfer 1, fatty acid transporter, beta hydroxyl butyric dehydrogenase etc.). Cluster analysis results showed that the up/down-regulated genes were highly responsive to RS treatment, and were considered to be directly or indirectly associated with reducing plasma glucose and body fat. To interpret the mechanism of RS regulation at the molecular level, a novel gene network was constructed based on 370 up/down-regulated genes coupled with 718 known diabetes-related genes. The topology of the network showed the characteristics of small-world and scale-free network, with some pathways demonstrating a high degree. Forkhead class A signaling pathway, with a degree of 8, was analyzed and was found to have an effect mainly on glucose and lipid metabolism processes. The results indicate that RS can suppress the development of type 2 diabetes in the STZ rat model through modulating the expression of multiple genes involved in glucose and lipid metabolism. The potential application of this novel gene network is also discussed.

**Electronic supplementary material:**

The online version of this article (doi:10.1186/s40064-015-0873-2) contains supplementary material, which is available to authorized users.

## Introduction

Starch is the most abundant storage glucan composed of two main structural components, amylose and amylopectin. Based on the rate of digestion, starch can be classified as rapidly digestible starch (RDS), slowly digestible starch (SDS), and resistant starch (RS) (Shi and Maningat 2013). In recent years, the effect of RS has drawn increasing interests in that it is not digested in the upper gastrointestinal tract, but is fermented in the large intestine and is beneficial for the gut environment (Lafiandra et al. 2014). Our recent research has shown that the applications of RS in foods can moderate the glycemic response and maintain a proper microorganism profile in the human gut (Zhou et al. 2013a, b). It has also been found that RS with a slow absorption and a low glycemic index can reduce blood lipids and improve insulin sensitivity, which can improve the glucose intolerance and insulin resistance (Goda et al. 2014). Previous studies have also shown that RS can reduce cholesterol and triglyceride levels in liver and serum of type 2 diabetic rats (Britto et al. 2004).

Diabetes affects approximately 8% of the United States population and 23% of the population >60 y of age, manifested predominantly as type 2 diabetes (von der Schulenburg and Frank 2014) and treatment for diabetes is relatively limited with significant side effects. Previous reports have indicated that lifestyle modifications, such as increasing fasting and exercise may reduce some risk factors such as postprandial glucose levels, decreased insulin sensitivity and obesity, which are more effective than pharmacological intervention in delaying the onset of type 2 diabetes (Stanhope et al. 2009). The discovery of natural products with health benefits as an alternative approach to current medications is the focus of intensive research has become many people’s interest. To investigate the contributing role of RS in maintaining normal plasma glucose and body fat levels, we have used a rat model of type 2 diabetes mellitus as a pathological model.

Analysis of gene expression variation in the liver, the site of lipid degradation and glycogen storage, is a widely used approach to understand blood glucose and body fat level changes (Kodama et al. 2012). To discover new genes or new molecular networks involved in the diabetes pathogenesis, microarray technology currently offers the fastest and most comprehensive molecular evaluation (Dillies et al. 2013) and many studies have applied microarrays to understand gene expression profiles in Diabetes Mellitus (Lowe et al. 2007; del Rosario et al. 2014). However, limited reports have described that functional foods or materials directly modulated gene expression profiles of diabetes-relevant organs (Park et al. 2012). Thus, in this study, genome-wide analyses are performed in liver tissues of STZ-induced diabetic rats before and after RS treatment using an oligonucleotide microarray.

## Materials and methods

### Materials

RS, from high amylose maize (Hi-maize™), was obtained from National Starch and Chemical Company, NSW, Australia. Streptozotocin (STZ) was purchased from Sigma-Aldrich (St. Louis, MO, USA). Other chemicals were of reagent grade and used as received.

### Animals and diets

Healthy male Sprague–Dawley rats (non-diabetic) of 190 ± 10 g weight were purchased from the animal house, Chinese Military Medical Science Academy. 16 rats were divided into two groups randomly: model control and RS interventional group. SD rats were housed in plastic cages (4 rats/cage) with free access to food and water, under controlled conditions of humidity (55 ± 5%), light (12/12 h light/dark cycle) and temperature (at 23°C). After one week’s adaptive feeding with the basic diet, the rats were fasted for 12 h, followed by intravenous injection of STZ 45 mg/ kg except the rats in normal control group. After 72 h of the injection, the fasting blood glucose (FBG) levels were estimated using a glucometer (ACCU-CHEK, ROCHE). Rats with FBG levels higher than 16.7 mmol/L were considered diabetic and were included insubsequent experiments. Normal control and model control continued with the basal diet for 4 weeks. RS was administered for the interventional group by oral gavage, using a feeding needle with 2 g once daily (which was about 8% of the total diet) for 4 consecutive weeks before the animals were sacrificed. There were no obvious signs of toxicity throughout the course of the experiments and all treated animals survived. The basal diet contained 7% fat, 13% protein, and a highly digestible starch. During the experimental course of the 4 weeks, blood samples were collected from the tail vein and body weights were recorded weekly (Table 1). Experimental procedures were approved by the Animal Ethics Committee of PLA Military Science and complied with the Chinese Code of Practice for the Care and Use of Animals for Scientific Purposes.

**Table 1:** **Change in the body weights of type 2 diabetic rats following the RS treatment**

	**Body weights (g)**					
**Group**	**Initial weight**	**72 h**	**1 week**	**2 weeks**	**3 weeks**	**4 weeks**
Model control	204.60 ± 7.83	201.20 ± 9.42	202.60 ± 9.32	181.20 ± 14.25	216.20 ± 13.07	204.60 ± 18.24
RS treatment	201.80 ± 6.75	201.00 ± 7.32	203.00 ± 9.06	195.20 ± 8.52	213.30 ± 8.10	213.80 ± 9.92

Results were expressed as means ± SD (n = 8, one-way ANOVA). There was no significant difference in the body weights of the diabetic rats between the two groups.

### Biological analysis

At the end of the experimental feeding period (4 weeks), blood samples were collected from the femoral artery after sacrifice and stored at −80°C prior to chemical analyses. Blood lipid composition including high-density lipoprotein-cholesterol (HDL-c), total cholesterol (TCH) and triglyceride (TG), were measured according to the kit instructions (Jiancheng Biological Engineering Institute, Nanjing, China).

After the 4-weeks feeding, the rats were dissected immediately with sterile scissors. The livers were sampled, weighed and immediately placed in liquid nitrogen until frozen thoroughly. All the liver samples were then stored at −80°C until RNA extraction.

### Microarray design

Liver tissue was removed from storage and homogenized. The total RNA was extracted using the Trizol reagent (Takara) and then treated with RNase-free DNase to remove contaminating genomic DNA. The quality and integrity of RNA was assessed using agarose gel electrophoresis. The microarray analysis of samples for RS (for RS treatment) and CK (for comparison) was performed by the Bioassay Laboratory of CapitalBio Corporation using the Rat Genome 230 2.0 Array (Affymetrix). Quality control parameters, positive control signals such as Oligo B2, Poly-A Controls and Hybridization Controls were all normal. The house-keeping gene signals and the ratio of 3′ on 5′ also remained normal with low average background values and noise values.

### Comparison analysis

The image signal data were first stored as a . DAT file, and then transformed to digital signal data as a .CEL file using AGCC software (Affymetrix Gene Chip Command Console Software). The fluorescence signal intensity levels were pre-analyzed using the RMA algorithm (Irizarry et al. 2003) for gene expression comparison analysis: 3 or more biological replications were performed and analyzed, using the R language package based on SAM (significance analysis of microarray) to analyze the differential gene expression, the screening standard was: Q-value ≤ 5% and the Fold Change ≥ 2 or ≤ 0.5.

### Statistical analysis

The results were analyzed for statistical significance by one-way analysis of variance (ANOVA) test using the Statistical Package of the Social Science (SPSS) program. All data are expressed as mean ± SD values. In all analyses, a *P* < 0.05 was considered statistically significant.

## Results and discussion

### Gene expression profile in the liver tissues of diabetic rats

Prior to the gene expression study, the effect of RS on plasma glucose and body fat levels was determined in STZ-induced diabetic rats. Our previous results revealed that rats treated with RS for two-week’s showed a 15.9% reduction in blood glucose levels compared to its initial level, and there was a total 27.9% reduction in the blood glucose level after 4-weeks treatment of RS, resulting in a significant difference compared to the model control group (*P* < 0.001) (data submitted for publication).

**Figure 1:**
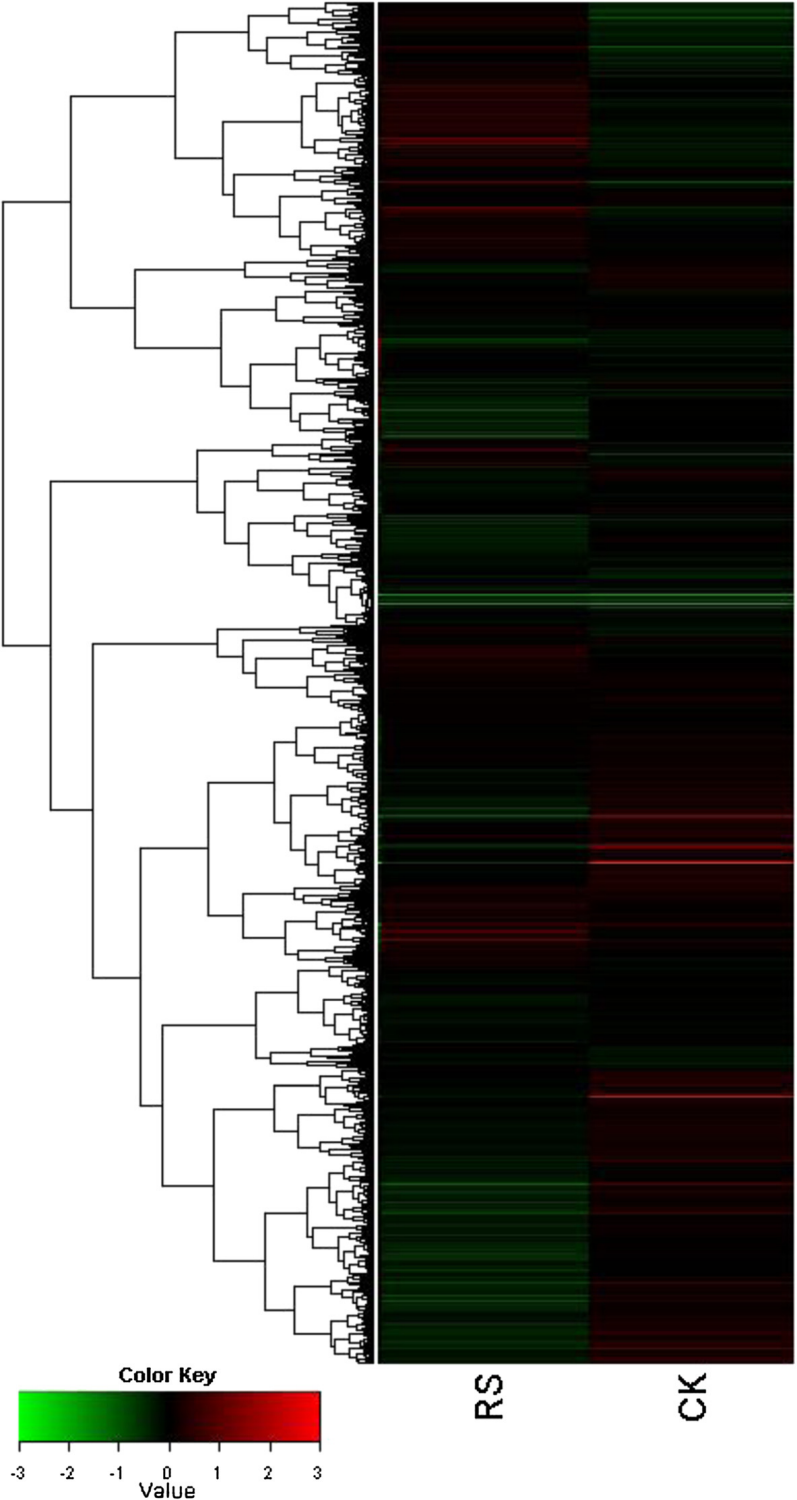
**Cluster analysis of genes before (CK) and after RS treatment.**

Gene expression profiling was carried out using the rat genome 230 2.0 array (Affymetrix Cooperation) in liver tissues of STZ-induced and RS-treated STZ-induced diabetic rats. Hierarchical clustering was used to globally assess the function-dependent gene expression patterns and initial interpretation of the extensive data set, and in particular, to identify correlated expression patterns that reflect the biological processes occurring in the pancreatic tissues. As shown in Figure 1, clustering display highlights the differential expression of numerous genes (up- or down regulated) among the groups. Among the 31,041 total genes, exactly 370 were counted as differentially expressed genes (ratio > 2 or <0.5) in the rat livers. Among them, 173 genes were up-regulated by RS treatment, and 197 genes were down-regulated. These results suggest that liver-specific gene expression is responsive to RS treatment, and this expression pattern could be considered to be directly or indirectly associated with reducing blood glucose and body fat activity.

### Local gene network and topology analysis

To interpret the effect of RS on diabetic physiological indices, specific genes associated with diabetes were analyzed using the Rat Genome Database (Simon et al. 2007). The significantly regulated diabetes-associated genes from the rat genome database were presented in Table 2. A total of 17 genes were observed to be differentially expressed in the liver in the RS treatment group compared with STZ-induced diabetic rats without treatment. Specifically, this included 7 up-regulated genes (*Cpt1a, G6pc, Txnip, Hmgcr, Src, Pfas, Ppargc1b*) and 10 down-regulated genes (*Ccl2, Serpina7, Fga, Pik3c2a, Isg15, Gck, Casp12, Cxcl10, Apln, Kng1*).

**Table 2:** **Diabetes-associated genes up/down-regulated in the liver of diabetic rats after RS treatments**

**Gene symbol**	**Gene name**	**Ratio**
Cpt1a	carnitine palmitoyltransferase 1a, liver	2.2353
G6pc	glucose-6-phosphatase, catalytic subunit	2.3547
Txnip	thioredoxin interacting protein	2.1232
Hmgcr	3-hydroxy-3-methylglutaryl-CoA reductase	2.0119
Src	SRC proto-oncogene, non-receptor tyrosine kinase	2.102
Pfas	Phosphoribosylformylglycinamidine synthase	2.524
Ppargc1b	peroxisome proliferator-activated receptor gamma, coactivator 1 beta	2.6752
Ccl2	chemokine (C-C motif) ligand 2	0.3036
Serpina7	serpin peptidase inhibitor, clade A (alpha-1 antiproteinase, antitrypsin),member 7	0.3717
Fga	fibrinogen alpha chain	0.3675
Pik3c2a	phosphatidylinositol-4-phosphate 3-kinase, catalytic subunit type 2 alpha	0.452
Isg15	ISG15 ubiquitin-like modifier	0.2822
Gck	glucokinase	0.3975
Casp12	caspase 12	0.4165
Cxcl10	chemokine (C-X-C motif) ligand 10	0.4329
Apln	apelin	0.4918
Kng1	kininogen 1	0.4918

**Figure 2:**
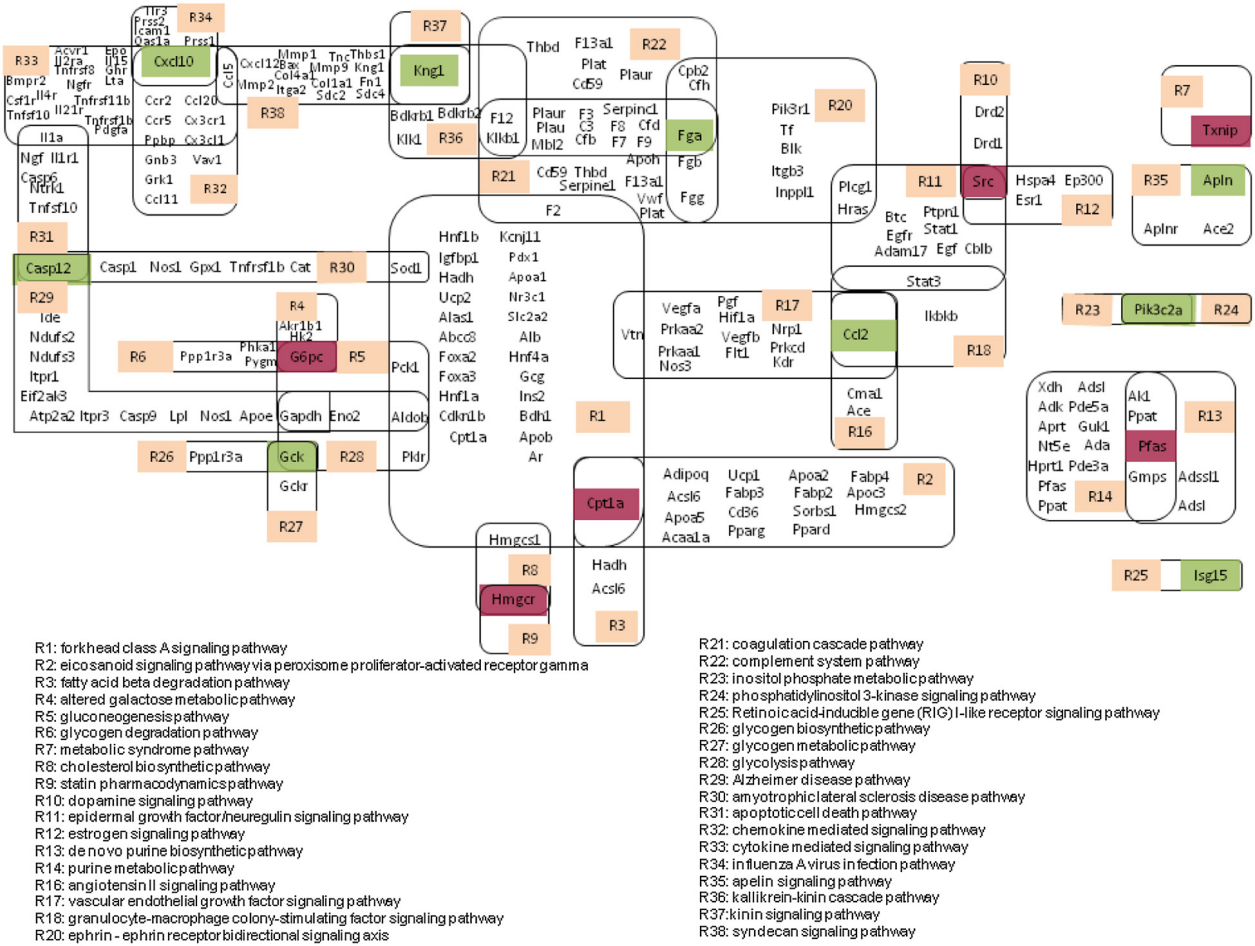
**Local gene networks (LGN) of diabetes-associated up/down-regulated genes connected by indicated pathways.** The genes with red base were up-regulated in response to RS treatment, while those with a green base were down-regulated. Other highlighted genes were diabetes-associated genes which participated in the pathways.

The diabetes-associated genes that participate in the same biological pathways as those differentially expressed by RS treatment were investigated simultaneously. These genes were collected and arranged, and a Local Gene Network (LGN) was produced as shown in Figure 2, where most pathways were connected with diabetes-related genes that were differently expressed or not. The genes that were up/down regulated were labeled with red/green base, respectively. There were 38 pathways involved in the connection of diabetes-related up/down regulated genes. Among them, 31 pathways were included in a large main network, while 7 other pathways were isolated from the main network.

From a topological perspective where a gene is commonly reported participating in about 3 pathways, the average degree of the network is approximately 3.0. However, some main pathways show larger degrees than others, such as the R1-forkhead class A signaling pathway has a degree of 8. As observed, the main network showed the characteristics of a small-world network, in terms of the average path length, some of which very short with a value of 3.68. In addition, a scale-free network feature was also clear and from the hierarchical structures can be seen in several local motifs, overall the cluster coefficient of the main network was 0.26.

### Forkhead class A signaling is a core pathway in the network

In the LGN (Figure 2), it is clearly demonstrated that R1-forkhead class A signaling pathway is connected with 8 pathways (R2-eicosanoid signaling pathway, R3-fatty acid beta degradation pathway, R5-gluconeogenesis pathway, R8-cholesterol biosynthetic pathway, R17-vascular endothelial growth factor signaling pathway, R21-coagulation cascade pathway, R28-glycolysis pathway, R30-amyotrophic lateral sclerosis disease pathway). These pathways are connected directly with the main glucose metabolism and lipid metabolism processes. Forkhead family, a group of conserved transcription factors in both yeast and human, is distinguished by the presence of a 100aa forkhead DNA binding domain. Forkhead transcription factors play important roles in regulating a number of cell processes including development, cell proliferation and differentiation, maintenance of stemness, stress response, language acquirement, and longevity (Tuteja and Kaestner 2007). Among the genes described in this pathway, *Igfbp1* and *Hnf4a* also present in the hypoxia inducible factor HIF-1 pathway (Steuerwald et al. 2010). HIF-1-regulated target genes include erythropoietin (EPO), vascular endothelial growth factor (VEGF), insulin-like growth factor II (IGF-2) and examples of glycolytic enzymes (aldolase A, enolase 1, 1actate dehedrogenase A, phosphofructokinase L, phosphoglycerate kinase1, glyceraldehydes-3-phosphate dehydrogenase, etc.). The *Hadh* gene regulates 3-hydroxyacyl-CoA dehydrogenase (Kapoor et al. 2009), and is reported mainly to participate in the metabolism of fatty acids, in particular the 3^rd^ process of *β*-oxidation. These results may indicate that this pathway could be regulated by glucose and lipid metabolism in response to RS treatment.

### Glucose and lipid metabolism modules analysis

**Figure 3:**
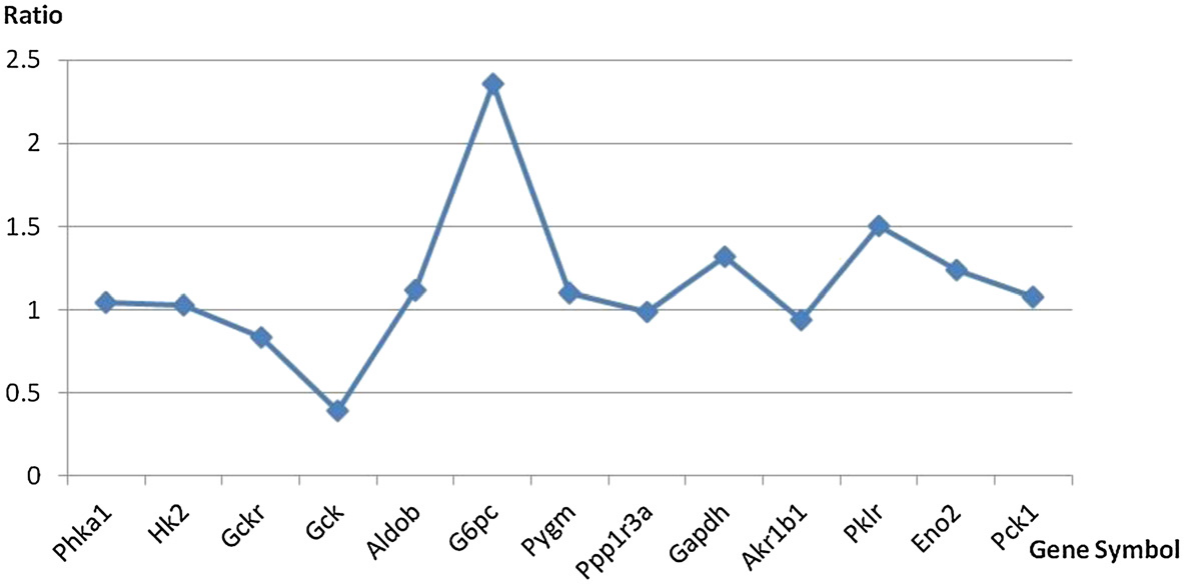
**Ratio of the expression of the glycol-metabolism-related genes before and after RS treatment.**

From these results a close relationship between the forkhead class A signaling pathway and glucose and lipid metabolism could be observed (Figure 2). 13 genes related to glucose metabolism in the LGN were sequenced by the order they appeared in the metabolism process (Table 3), and two genes were differently expressed in response to RS treatment. The ratios of the expression of the glucose metabolism-related genes after and before RS treatment were shown in Figure 3. The majority of the glucose metabolism-related genes were shown to be up-regulated with RS treatment (Ratio > 1), indicating that the glucose metabolism process becomes more active with the addition of RS, which may result in a decrease in blood glucose levels. Conversely, down-regulated genes did not express key enzymes included in glucose metabolism. More specifically, the genes related to glycolysis were up-regulated in varying degrees (*Hk2*, *Pklr*, *Gapdh,* etc.). The genes which were down-regulated, such as *Gck*, were not involved in glycolytic process.

**Table 3:** **Glucose metabolism-related genes in the LGN**

**Gene symbol**	**Gene name**
Phka1	phosphorylase kinase, alpha 1
Hk2	hexokinase 2
Gckr	glucokinase (hexokinase 4) regulator
Gck	glucokinase
Aldob	aldolase B, fructose-bisphosphate
G6pc	glucose-6-phosphatase, catalytic subunit
Pygm	phosphorylase, glycogen, muscle
Ppp1r3a	protein phosphatase 1, regulatory subunit 3A
Gapdh	glyceraldehyde-3-phosphate dehydrogenase
Akr1b1	aldo-keto reductase family 1, member B1 (aldose reductase)
Pklr	pyruvate kinase, liver and RBC
Eno2	enolase 2, gamma, neuronal
Pck1	phosphoenolpyruvate carboxykinase 1 (soluble)

The genes were sequenced in the order they appeared in the metabolism process.

**Figure 4:**
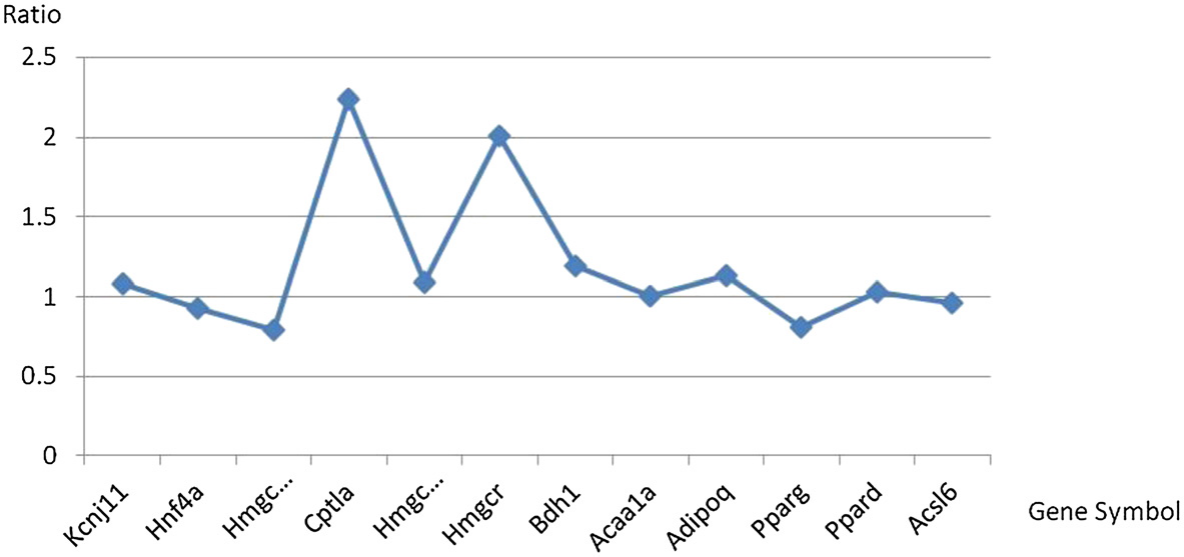
**Ratio of the expression of the lipid metabolism-related genes before and after RS treatment.**

Using a similar approach, the 12 genes related to lipid metabolism in the LGN were sequenced according to the order they appeared in the metabolism process (Table 4), and two genes were over-expressed in response to RS treatment. The ratio of expression of the lipid metabolism-related genes after and before RS treatment was shown in Figure 4. Similar to the results associated with glucose metabolism, most of the lipid metabolism-related genes were up-regulated with RS treatment (Ratio > 1), which also indicated that the lipid metabolism processes may become more active with the addition of RS, which may result in a decrease in body fat.

**Table 4:** **Lipid metabolism-related genes in the LGN**

**Gene symbol**	**Gene name**
Kcnj11	potassium inwardly rectifying channel, subfamily J, member 11
Hnf4a	hepatocyte nuclear factor 4, alpha
Hmgcs1	3-hydroxy-3-methylglutaryl-CoA synthase 1 (soluble)
Cptla	carnitine palmitoyltransferase 1a, liver
Hmgcs2	3-hydroxy-3-methylglutaryl-CoA synthase 2 (mitochondrial)
Hmgcr	3-hydroxy-3-methylglutaryl-CoA reductase
Bdh1	3-hydroxybutyrate dehydrogenase, type 1
Acaa1a	acetyl-CoA acyltransferase 1
Adipoq	adiponectin, C1Q and collagen domain containin
Pparg	peroxisome proliferator-activated receptor gamma
Ppard	peroxisome proliferator-activated receptor delta
Acsl6	acyl-CoA synthetase long-chain family member 6

The genes were sequenced in the order they appeared in the metabolism process.

In this study, we have proposed a novel gene network based on a comparative genome-wide analysis and used a knowledge-based gene assembly to analyze the possible molecular mechanisms of the RS effect. Some pathways were not connected to the main network possibly because not all diabetes-related genes have been well reported. As a high-throughput method, microarray technology may be not fully accurate in that not all target sites were accurately tested. In this study, a genome-wide analysis was performed where the main characteristics of the network could be universal. In terms of the network topology, alterations with glucose and lipid metabolism networks were consistent with the proposed effects of RS as a dietary intervention, suggesting that this analytical method using a recombination-based network could be used for the analysis of similar substances. However, further work is needed to clarify the molecular mechanisms of RS in reducing the levels of plasma glucose and body fat.

## Conclusion

In this study, we demonstrate that RS may provide direct signals to rat liver cells and regulate the expression of a number of diverse genes, suggesting that RS may modulate its effect through a network involving complex gene regulatory events. These altered levels of gene expression in response to RS treatment may indicate that the candidate genes and their surrounding network partners could contribute to the array of complications in the diabetic mammals. Importantly, we show that the bioinformatics analysis is a feasible and powerful approach to interpret the underlying molecular mechanism of RS. Further study is required to further elucidate the pathways affected by RS treatment and this will be further enhanced by increased annotation of the rat genome.
